# Nonpharmaceutical interventions and postpandemic resurgence: the shifting epidemiology of influenza, respiratory syncytial virus, and adenovirus in Hangzhou, Zhejiang Province (2020–2024)

**DOI:** 10.1093/femsmc/xtag040

**Published:** 2026-07-17

**Authors:** Shaoni Zhang, Shilei Dong, Yan Qi, Yanjing Shen, Yanhong Sun

**Affiliations:** Department of Clinical Laboratory, Hangzhou Traditional Chinese Medicine Hospital Affiliated to Zhejiang Chinese Medical University, Hangzhou 310012, China; Department of Clinical Laboratory, Zhejiang Hospital, Hangzhou 310013, China; Department of Clinical Laboratory, Hangzhou Traditional Chinese Medicine Hospital Affiliated to Zhejiang Chinese Medical University, Hangzhou 310012, China; Department of Clinical Laboratory, Hangzhou Traditional Chinese Medicine Hospital Affiliated to Zhejiang Chinese Medical University, Hangzhou 310012, China; Department of Clinical Laboratory, National Clinical Research Center for Child Health, National Children’s Regional Medical Center, Children’s Hospital, Zhejiang University School of Medicine, Hangzhou 310052, China

**Keywords:** age distribution, COVID-19 pandemic, epidemiological surveillance, NPIs, respiratory viruses, seasonal patterns

## Abstract

Acute respiratory infections caused by influenza A/B (Flu A/Flu B), respiratory syncytial virus (RSV), and adenovirus (ADV) remain a major global health burden. The COVID-19 pandemic profoundly altered respiratory virus transmission through nonpharmaceutical interventions (NPIs). Samples from patients with respiratory tract infections at Hangzhou Hospital of Traditional Chinese Medicine from January 2020 to October 2024 were collected. ADV, Flu A, Flu B, and RSV were detected to evaluate the impact of NPIs on viral epidemiology. Statistical analyses were performed using SPSS software and GraphPad Prism. Multivariable binary logistic regression models, adjusting for age group and sex, were constructed to evaluate the independent effect of NPI relaxation (strict NPI period 2020-2022 vs. post-relaxation period 2023–2024) on virus positivity. Stratified analyses by age group and demographic comparability assessments between periods were also performed. Overall virus positivity declined from 27.4% (2020) to 14.5% (2021) during strict NPIs, then rebounded to 35.1% in 2023 after relaxation. Flu A resurged atypically in summer 2022 and dominated the 2023 winter peak, while Flu B increased annually and peaked in 2024. ADV regained its prepandemic summer seasonality by 2024, whereas RSV showed limited seasonal variation. Higher ADV positivity was observed in males, and higher Flu B positivity in females. By 2024, influenza burden shifted toward adults and geriatric populations, while children under five remained most vulnerable to RSV and ADV. However, after adjusting for demographic shifts—including a significant increase in adult patients (18–60 years: 22.1%–40.0%) and altered sex ratios between periods (both *P* < 0.001)-the post-relaxation period showed lower adjusted odds of positivity for all four pathogens (Flu A: aOR = 0.64, 95% CI: 0.61–0.67; Flu B: aOR = 0.67, 95% CI: 0.63–0.70; ADV: aOR = 0.72, 95% CI: 0.68–0.76; RSV: aOR = 0.62, 95% CI: 0.59–0.65; all *P* < 0.001), whereas no significant sex differences were observed after multivariable adjustment (all *P* > 0.05). Stratified analyses further revealed that the magnitude of period-related risk reduction increased with age for all pathogens. This study demonstrates that the postpandemic resurgence of respiratory viruses in Hangzhou, Zhejiang Province, was not a monolithic recrudescence following NPIs relaxation, but a multifaceted shifting epidemiology driven by three convergent forces: expanded school-based and community screening that broadened the tested denominator, demographic case-mix shifts toward higher-risk adult populations, and pathogen-specific alterations in seasonal and age-specific transmission patterns. The consistently lower adjusted odds of positivity during the post-relaxation period indicate that crude resurgence signals must be interpreted through the lens of surveillance structure. This study demonstrates that the postpandemic resurgence of respiratory viruses in Hangzhou (2020–2024) was not a uniform transmission rebound following NPI relaxation, but a shifting epidemiology shaped by surveillance structural changes, pathogen-specific resilience, and altered population immunity. Multivariable models showed lower adjusted odds after restriction easing, while age-stratified analyses revealed heterogeneous period effects superimposed on stable risk hierarchies. In this subtropical Chinese city, influenza reasserted prepandemic seasonality, whereas RSV and ADV exhibited delayed recovery. These insights advocate for adaptive, age-targeted public health strategies-prioritizing adult vaccination for influenza and sustained pediatric prevention for RSV and ADV-that account for both biological transmission dynamics and surveillance artifacts in the post-COVID-19 era.

## Teaser

Epidemiological surveillance of respiratory viruses in Hangzhou, China (2020-2024) revealed profound alterations in the epidemiology of influenza, adenovirus, and Respiratory Syncytial Virus due to Nonpharmaceutical Interventions.

## Introduction

Acute respiratory infections (ARIs) are a leading cause of global hospitalization and mortality worldwide, particularly among pediatric, immunocompromised, and geriatric populations (Levine et al. 2022). A nationwide surveillance study in China reported viral and bacterial positivity rates of 46.9% and 30.9%, respectively, among children under 5 years old and school-age children. Influenza virus, respiratory syncytial virus (RSV), and human rhinovirus (HRV) were the most common viruses, accounting for 28.5%, 16.8%, and 16.7% of detections, respectively (Wu et al. 2023). Pathogen prevalence varies across age groups, although Flu A and HRV are consistently among the most frequently detected viruses (Chen et al. 2026). Geriatric populations are at greater risk of respiratory viral infections because of immunosenescence and inflammaging (Huang et al. 2025, Fragkou et al. 2026). Because definitive etiological diagnosis remains challenging in routine clinical practice, robust surveillance programs are needed to guide vaccine deployment, optimize healthcare resources, and control outbreaks (Gaitonde et al. 2019).

Seasonal influenza epidemics are primarily caused by Flu A/B viruses, resulting in an estimated 1 billion annual cases and 290 000–650 000 respiratory-related deaths worldwide; 99% of pediatric fatalities occur in developing countries (Wang et al. 2020, Liang et al. 2023). RSV is the leading cause of lower respiratory tract infections in infants (González et al. 2012). In 2019, RSV was associated with an estimated 3 million hospitalizations and 59 600 in-hospital deaths among children under 5 years old globally (Li et al. 2022, Nguyen-Van-Tam et al. 2022). Since the COVID-19 pandemic, surveillance has shown a marked increase in adenovirus (ADV) detection in severe pediatric pneumonia, making it a major cause of severe community-acquired pneumonia in children under 5 years old (Chen et al. 2014, Radke et al. 2018, Lynch et al. 2021). However, these descriptive comparisons are potentially confounded by concurrent shifts in healthcare-seeking behavior, testing policies, and demographic case-mix between the nonpharmaceutical interventions (NPIs) and post-NPIs periods. For example, expanded school-based and community screening after 2023 may have broadened the tested population beyond the symptomatic, pediatric-predominant cohort of the earlier period, thereby complicating direct comparisons of crude positivity rates. Consequently, multivariable approaches adjusting for age, sex, and other demographic factors are required to isolate the independent effect of policy relaxation on viral epidemiology.

During the COVID-19 pandemic, NPIs substantially altered respiratory virus transmission patterns (Zhu et al. 2020, Angoulvant et al. 2021, Liu et al. 2021). Several regions analyzed postpandemic viral infection trends and identified distinct epidemiological characteristics (Wei et al. 2024, Li et al. 2024, Zhao et al. 2024). To characterize trends in Hangzhou, researchers evaluated respiratory virus test results from patients with influenza-like illness at a tertiary hospital from 2020 to 2024 to clarify province-specific epidemiological patterns of ARIs. We employed multivariable binary logistic regression models to assess period-specific virus positivity while adjusting for demographic confounders, complemented by stratified analyses to examine age-dependent effect modification. These analyses aim to clarify Hanghzou-specific epidemiological patterns of ARIs in the postpandemic era and inform adaptive public health strategies.

## Methods

### Specimen collection and grouping

Nasopharyngeal or oropharyngeal swab specimens were collected from outpatients and inpatients from January 2020 to October 2024. All eligible patients tested during the study period were included in the analysis. The hospital is located in Hangzhou, the capital of Zhejiang Province, on the southeastern coast of China, which has a subtropical monsoon climate with four distinct seasons.

The inclusion criteria were as follows: (1) Symptoms consistent with ARIs, including fever ≥ 37.3°C, cough, sore throat, rhinorrhoea, nasal congestion, wheeze, or dyspnoea. (2) Onset-to-presentation interval ≤ 7 days and no prior antiviral or antibiotic therapy.(3) Age 1 day-100 years; either sex. (4) Informed consent obtained from the patient or legal guardian and willingness to provide naso/oropharyngeal swabs. (5) Complete clinical data, including age, sex, residential address, and symptom-onset date.

The exclusion criteria were as follows: (1) Repeated sampling: defined as multiple swabs from the same patient within 7 days of the same illness episode, in which only the first specimen was retained. (2) Clear noninfectious aetiology such as allergic rhinitis, acute asthma exacerbation, AECOPD. (3) Specimens collected ≥ 48 h after hospital admission. (4) Poor sample quality including dry swabs or transport time > 4 h without a cold chain. (5) Inability to cooperate because of neuropsychiatric disorders.

Patients were classified into four age groups: infants and young children (0–<5 years old), school children (5–<18 years old), adults (18–<60 years old), and geriatric populations (≥60 years old). Seasons were defined as winter (December to February), spring (March to May), summer (June to August), and autumn (September to November).

### Respiratory pathogens detection

Nasopharyngeal swabs were collected by nurses into sampling tubes prefilled with 0.5 ml of extraction buffer and squeezed for 10–15 seconds to release viral antigens. Samples were immediately sent to the hospital Medical Laboratory Center for testing. Respiratory pathogens, including Flu A, Flu B, RSV, and ADV were detected using the colloidal gold method (Kaibili respiratory virus antigen detection kit, Hangzhou Genesis Corporation) according to the manufacturer’s instructions. Results were available within 15 min. Samples were considered positive when both the control and test lines changed color. If the control line failed to change color, the result was considered invalid and retesting was rquired. Coinfection was defined as the simultaneous detection of two or more respiratory viruses, including Flu A, Flu B, RSV, and ADV, in a single patient.

### Statistical analysis

SPSS version 26.0 was used for statistical analysis, and GraphPad Prism version 9.5.0 was used for data visualization. Categorical variables were presented as counts, proportions (%), and positivity rates (%). Because age was not normally distributed, the median was used for analysis. Pearson’s chi-square test and Fisher’s exact tests were used for significance testing. *P* values < 0.05 were considered statistically significant.

To evaluate the independent effect of pandemic policy relaxation (strict NPI period [2020–2022] vs. post-relaxation period [2023–2024]) on virus positivity while adjusting for demographic shifts between periods, multivariable binary logistic regression models were constructed for each pathogen, with age group and sex as covariates. Age-group-stratified analyses were also performed to examine potential effect modification by age. Given the large sample size, we emphasized effect sizes (adjusted odds ratios [aORs] with 95% confidence intervals [CIs]) over P values. Statistical significance was set at *P* < 0.05."

## Results

### Overall positive detection

**Figure 1:**
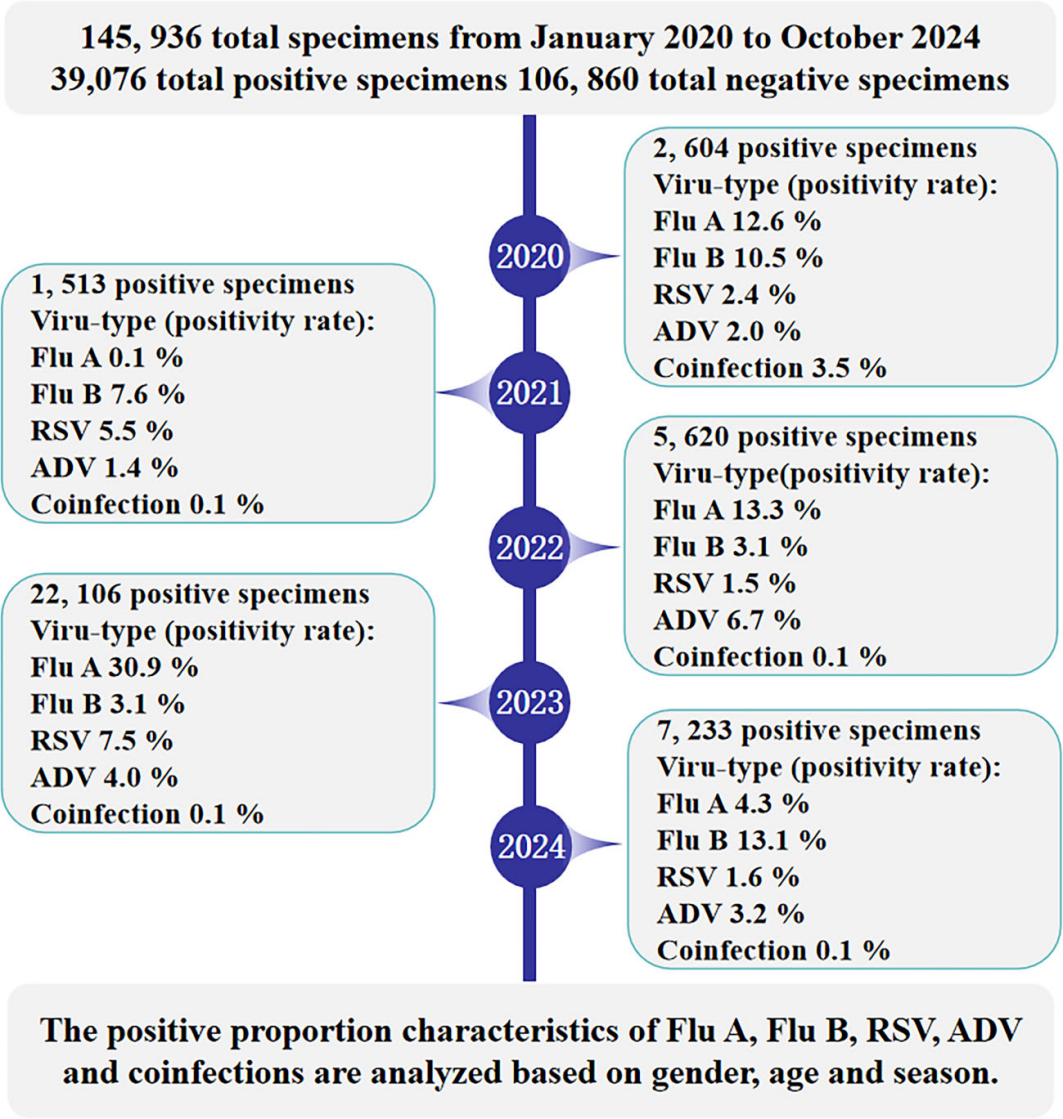
Flowchart of the methodology

A total of 145 936 specimens from patients with ARIs were tested from 2020 to 2024(Fig. 1). Among 145 936, 39 076 specimens tested positive for at least one virus, yielding an overall positivity of 26.8%. Of the positive cases, 26 101 were Flu A-positive, 8 911 Flu B-positive, 2 243 RSV-positive, 1 821 ADV-positive, and 134 coinfections. Flu A was the most prevalent pathogen (17.9%), followed by Flu B (6.1%), RSV (1.5%), ADV (1.3%), and coinfections (0.1%) (Table 1).

**Table 1:** Demographic data and positivity rates of respiratory viruses over a five-year period.

Demographics	2020	2021	2022	2023	2024	Total	P value
Total positive specimens	2 604	1 513	5 620	22 106	7 233	39 076	<0.001*
Total negative specimens	6909	8933	24 807	40 802	25 409	106 860	
Total tested specimens	9 513	10 446	30 427	62 908	32 642	145 936	
Positivity rate	27.4%	14.5%	18.5%	35.1%	22.2%	26.8%	
Total							0.115
Male	1264/4545 (27.8%)	795/5552 (14.3%)	2962/15 749 (18.8%)	11 148/31 515 (35.4%)	3417/15 288 (22.4%)	19 586/72 649 (27.0%)	
Female	1340/4968 (27.0%)	718/4894 (14.7%)	2658/14 678 (18.1%)	10 958/31 393 (34.9%)	3816/17 354 (22.0%)	19 490/73 287 (26.6%)	
Flu A							0.1
Male	574/4545 (12.6%)	3/5552 (0.1%)	2131/15 749 (13.5%)	9777/31 515 (31.0%)	629/15 288 (4.1%)	13 114/72 649 (18.1%)	
Female	623/4968 (12.5%)	2/4894 (0)	1900/14 678 (12.9%)	9677/31 393 (30.8%)	785/17 354 (4.5%)	12 987/73 287 (17.7%)	
Flu B							0.014*
Male	455/4545 (10.0%)	431/5552 (7.8%)	481/15749 (3.1%)	957/31 515 (3.0%)	2000/15288 (13.1%)	4324/72649 (6.0%)	
Female	542/4968 (10.9%)	358/4894 (7.3%)	447/14 678 (3.1%)	972/31 393 (3.1%)	2268/17 354 (13.1%)	4587/73 287 (6.3%)	
RSV							0.244
Male	121/4545 (2.7%)	279/5552 (5.0%)	231/15 749 (1.5%)	260/31 515 (0.8%)	253/15 288 (1.7%)	1144/72 649 (1.6%)	
Female	103/4968 (2.1%)	294/4894 (6.0%)	227/14 678 (1.6%)	210/31 393 (0.7%)	265/17 354 (1.5%)	1099/73 287 (1.5%)	
ADV							<0.001*
Male	114/4545 (2.5%)	82/5552 (1.5%)	119/15 749 (0.8%)	153/31 515 (0.5%)	536/15 288 (3.5%)	1004/72 649 (1.4%)	
Female	72/4968 (1.5%)	64/5894 (1.1%)	84/14 678 (0.6%)	100/31 393 (0.3%)	497/17 354 (2.9%)	817/73 287 (1.1%)	
Coinfection							0.075
Male	15/4545 (0.3%)	5/5552 (0.1%)	13/15 749 (0.1%)	34/31 515 (0.1%)	10/15 288 (0.1%)	77/72 649 (0.1%)	
Female	18/4968 (0.3%)	3/4894 (0.1%)	9/14 678 (0.1%)	16/31 393 (0.1%)	11/17 354 (0.1%)	57/73 287 (0.1%)	
Virus detection, n (%)							
Flu A	1197(12.6%)	5(0.1%)	4031(13.3%)	19 454(30.9%)	1414(4.3%)	26 101(17.9%)	<0.001*
Flu B	997(10.5%)	789(7.6%)	928(3.1%)	1929(3.1%)	4268(13.1%)	8911(6.1%)	<0.001*
RSV	224(2.4%)	573(5.5%)	458(1.5%)	470(7.5%)	518(1.6%)	2243(1.5%)	<0.001*
ADV	186(2.0%)	146(1.4%)	203(6.7%)	253(4.0%)	1033(3.2%)	1821(1.3%)	<0.001*
Coinfection	33(3.5%)	8(0.1%)	22(0.1%)	30(0.1%)	21(0.1%)	134(0.1%)	<0.001*

Abbreviations: Flu A, influenza A; Flu B, influenza B; RSV, respiratory syncytial virus; ADV, adenovirus. *P < 0.05.

### Time trends of virus positivity for respiratory pathogens

**Figure 2:**
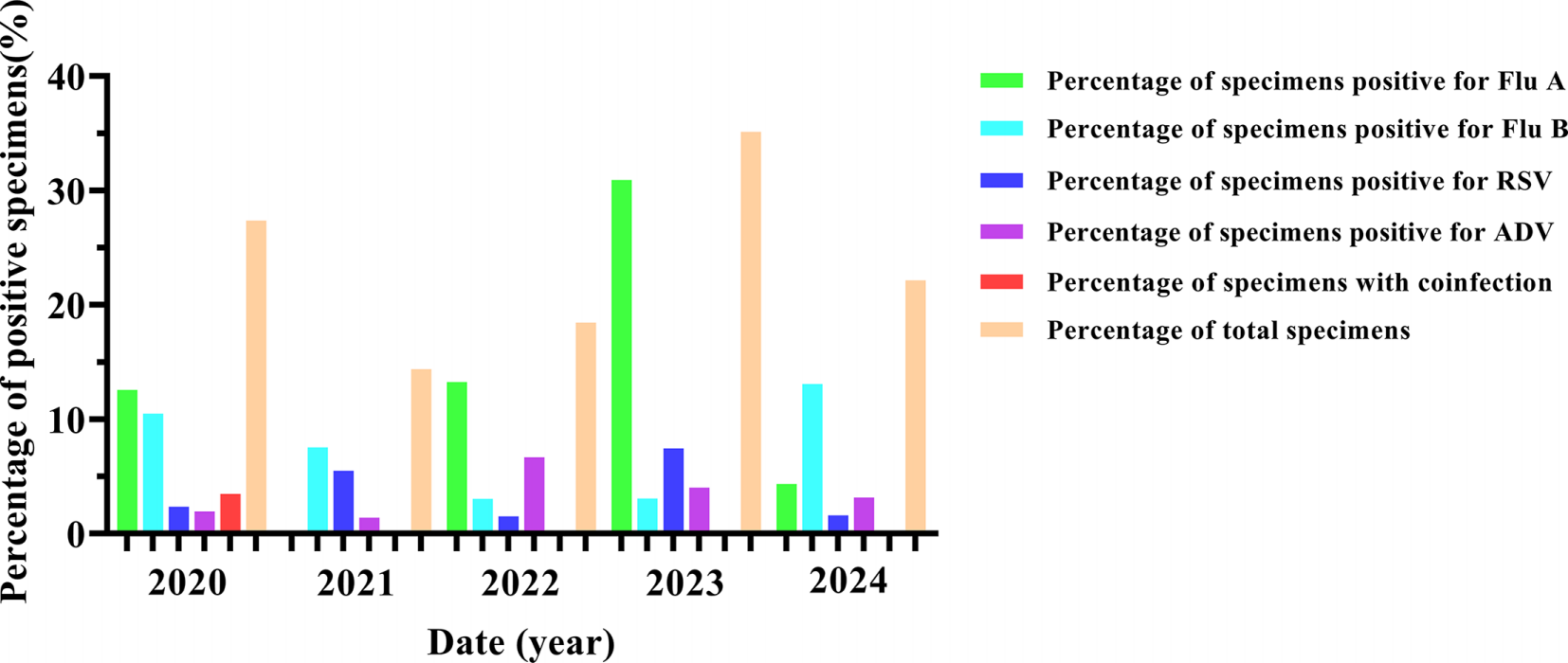
Positivity rates of Flu A, Flu B, RSV, ADV, and coinfections by year (2020–2024).

A chi-square test showed significant differences in positive specimens from 2020 to 2024 (*P* < 0.001) (Table 1). As shown in Fig. 2, the overall positivity rate declined from 27.4% in 2020 to 14.5% in 2021 and 18.5% in 2022, then peaked at 35.1% in 2023 before decreasing to 22.3% in 2024.

**Figure 3:**
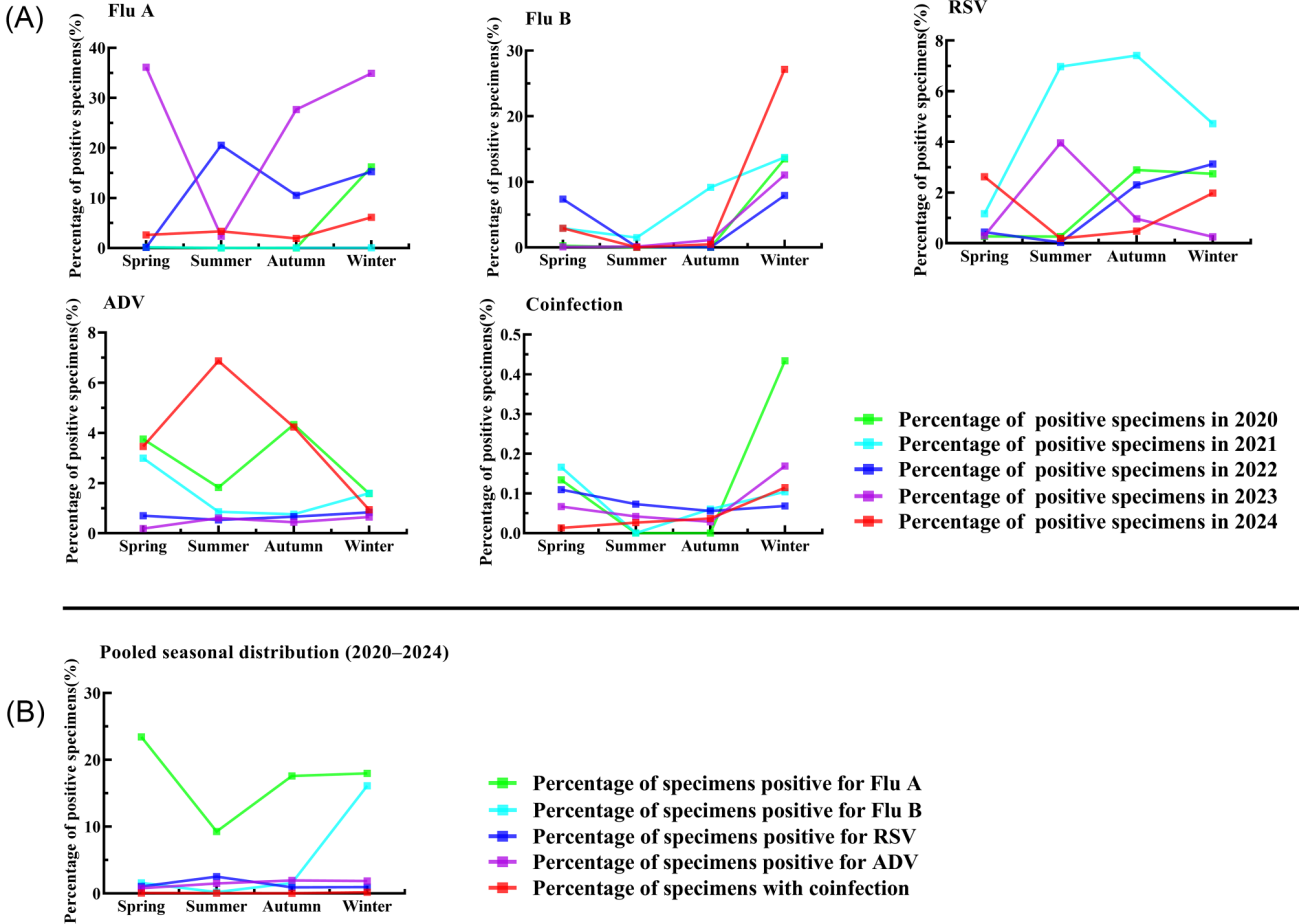
Seasonal patterns of four major respiratory viruses and coinfections from 2020 to 2024. (A) Seasonal distribution of positivity rates by year (2020–2024). Subpanels show Flu A, Flu B, RSV, ADV, and Coinfection. Each line represents one study year. (B) Pooled seasonal distribution (2020–2024). Each line represents the pooled seasonal distribution of positivity rates across the entire 5-year study period for Flu A, Flu B, RSV, ADV, and coinfections.

Table 2 and Fig. 3 show that respiratory infections increased markedly after NPIs were relaxed in 2023. Positivity rates rebounded for all pathogens, although trends differed among viruses. Flu A accounted for the largest proportion of cases and largely reflected the overall epidemic trend. Flu B declined from 2020 to 2023, with a slight increase in 2024. RSV positivity fluctuated without a sustained trend. ADV positivity declined in 2021, peaked in 2022, and decreased from 2023 to 2024. Coinfection positivity continuously declined throughout the study period.

**Table 2:** Comparison of the seasonal distribution of the number and positivity rates of five types of viral infections from 2020 to 2024.

	2020	2021	2022	2023	2024	Total	*P* value
**Flu A**							
Spring	1/746	3/1804	6/4571	9 179/25 417	197/7512	9 386/40 050	<0.001*
March to May	(0.1%)	(1.7%)	(0.1%)	(36.1%)	(2.6%)	(23.4%)	
Summer	0/766	0/2 453	1 964/9 570	113/4 776	253/7 562	2 330/25 127	<0.001*
June to August	(0)	(0)	(20.5%)	(2.4%)	(3.4%)	(9.3%)	
Autumn	0/623	1/3 306	943/8 959	4796/17 345	52/2 717	5 792/32 950	<0.001*
September to November	(0)	(0)	(10.5%)	(27.7%)	(1.9%)	(17.6%)	
Winter	1 196/7 378	1/2 883	1 118/7 327	5 366/15 370	912/14 851	8 593/47 809	<0.001*
December to February	(16.2%)	(0)	(15.3%)	(34.9%)	(6.1%)	(18.0%)	
**Flu B**							
Spring	2/746	53/1 804	337/4 571	26/25 417	220/7 512	638/40 050	<0.001*
March to May	(0.3%)	(2.9%)	(7.4%)	(0.1%)	(2.9%)	(1.6%)	
Summer	0/766	37/2 453	7/9 570	7/4 776	3/7 562	54/25 127	<0.001*
June to August	(0)	(1.5%)	(0.1%)	(0.2%)	(0.04%)	(0.2%)	
Autumn	0/623	303/3 306	3/8 959	198/17 345	13/2 717	517/32 950	<0.001*
September to November	(0)	(9.2%)	(0)	(1.1%)	(0.5%)	(1.6%)	
Winter	995/7 378	396/2 883	581/7 327	1 698/15 370	4 032/14 851	7 702/47 809	<0.001*
December to February	(13.5%)	(13.7%)	(7.9%)	(11.2%)	(27.2%)	(16.1%)	
**RSV**							
Spring	2/746	21/1 804	20/4 571	75/25 417	197/7 512	315/40 050	<0.001*
March to May	(0.3%)	(1.2%)	(0.4%)	(0.3%)	(2.6%)	(0.8%)	
Summer	2/766	171/2 453	3/9 570	189/4 776	14/7 562	379/25 127	<0.001*
June to August	(0.3%)	(7.0%)	(0)	(4.0%)	(0.2%)	(1.5%)	
Autumn	18/623	245/3 306	206/8 959	167/17 345	13/2 717	649/32 950	<0.001*
September to November	(2.9%)	(7.4%)	(2.3%)	(1.0%)	(0.5%)	(2.0%)	
Winter	202/7 378	136/2 883	229/7327	39/15 370	294/14 851	900/47 809	<0.001*
December to February	(2.7%)	(4.7%)	(3.1%)	(0.3%)	(2.0%)	(1.9%)	
**ADV**							
Spring	28/746	54/1 804	32/4 571	47/25 417	260/7 512	421/40 050	<0.001*
March to May	(3.8%)	(3.0%)	(0.7%)	(0.2%)	(3.5%)	(1.1%)	
Summer	14/766	21/2 453	51/9 570	29/4 776	519/7 562	634/25 127	<0.001*
June to August	(1.8%)	(0.9%)	(0.5%)	(0.6%)	(6.9%)	(2.5%)	
Autumn	27/623	25/3 306	59/8 959	77/17 345	115/2 717	303/32 950	<0.001*
September to November	(4.3%)	(0.8%)	(0.7%)	(0.4%)	(4.2%)	(0.9%)	
Winter	117/7 378	46/2 883	61/7 327	100/15 370	139/14 851	463/47 809	<0.001*
December to February	(1.6%)	(1.6%)	(0.8%)	(0.7%)	(0.9%)	(1.0%)	
**Coinfection**							
Spring	1/746	3/1 804	5/4 571	17/25 417	1/7 512	27/40 050	
March to May	(0.1%)	(0.2%)	(0.1%)	(0.1%)	(0)	(0.1%)	
Summer	0/766	0/2 453	7/9 570	2/4 776	2/7 562	11/25 127	
June to August	(0)	(0)	(0.1%)	(0)	(0)	(0)	
Autumn	0/623	2/3 306	5/8 959	5/17 345	1/2 717	13/32 950	
September to November	(0)	(0.1%)	(0.1%)	(0)	(0)	(0)	
Winter	32/7 378	3/2 883	5/7 327	26/15 370	17/14 851	83/47 809	
December to February	(0.4%)	(0.)	(0.1%)	(0.2%)	(0.1%)	(0.2%)	

Abbreviations: Flu A, influenza A; Flu B, influenza B; RSV, respiratory syncytial virus; ADV, adenovirus. *P < 0.05.

However, these crude trends do not account for concurrent shifts in the demographic structure of the tested population between the two periods (Table 3). When multivariable logistic regression was applied to adjust for age group and sex, the odds of positivity for all four pathogens were significantly lower during the post-relaxation period compared with the strict NPI period (all *P* < 0.001) (Table 4). Specifically, Flu A showed a 36% reduction in adjusted odds (aOR = 0.64, 95% CI: 0.61–0.67), followed by RSV (aOR = 0.62, 95% CI: 0.59–0.65), Flu B (aOR = 0.67, 95% CI: 0.63–0.70), and ADV (aOR = 0.72, 95% CI: 0.68–0.76).

**Table 3:** Demographic characteristics of the study population by study period.

Characteristic	Strict-NPI period (2020–2022), n (%)	Post-NPI period (2023–2024), n (%)	χ²	*P* value (Chi-square test)
**Age group**			427.4	<0.001*
<5 years	13 529 (26.8%)	13 248 (14.8%)		
5–<18 years	21 175 (42.0%)	31 700 (35.5%)		
18–<60 years	11 169 (22.1%)	35 720 (40.0%)		
≥60 years	4588 (9.1%)	8620 (9.7%)		
**Sex**			94.3	<0.001*
Male	24 508 (48.6%)	45 781 (51.3%)		
Female	25 953 (51.4%)	43 507 (48.7%)		

*χ² = Pearson chi-square test. *P* values derived from chi-square test for categorical variables.

**Table 4:** Results of multivariable logistic regression analyses for respiratory virus positivity.

Pathogen	Period aOR (95% CI)*	*P*	Age aOR (95% CI)*	*P*	Sex aOR (95% CI)*	*P*
Flu A	0.64 (0.61–0.67)	<0.001	2.86 (2.55–3.20)	<0.001	1.02 (0.97–1.06)	0.460*
Flu B	0.67 (0.63–0.70)	<0.001	2.40 (2.15–2.69)	<0.001	0.99 (0.95–1.04)	0.772
RSV	0.62 (0.59–0.65)	<0.001	2.06 (1.85–2.29)	<0.001	1.01 (0.97–1.06)	0.542
ADV	0.72 (0.68–0.76)	<0.001	2.33 (2.08–2.61)	<0.001	0.99 (0.94–1.03)	0.544

*aOR = adjusted odds ratio; CI = confidence interval. All models adjusted for age group and sex. The strict NPI period (2020–2022) served as the reference category for period; children aged <5 years served as the reference category for age group; and female served as the reference category for sex.

### Seasonal distribution of virus positivity for respiratory pathogens

Pearson’s χ² test showed significant seasonal variation in positivity rates for all viruses (*P* < 0.001) (Table 2). From 2020 to 2024, respiratory viruses showed distinct seasonal patterns. Flu A peaked in spring (23.4%) and winter (18.0%). After a marked decline in 2021 (five cases), positivity rebounded strongly in 2023 (36.1%), with winter-spring predominance re-emerging. Flu B also showed seasonal variation, peaking in spring (1.6%) and winter (16.1%). During the COVID-19 pandemic (2020–2022), Flu B positivity temporarily declined but rebounded from winter 2023 to winter 2024.

Compared with influenza viruses, RSV showed weaker seasonality, with higher positivity in autumn (2.0%) and winter (1.9%). ADV differed from the other viruses, with peak positivity occurring mainly in summer (2.5%) (Table 2). Seasonal peaks varied annually without a consistent pattern. Although coinfections were rare, they also showed seasonality, with the highest positivity in winter (0.2%), peaking in winter 2020 with 32 cases and a positivity rate of 0.4%. Figure 3 shows that influenza epidemics mainly occurred in winter and spring, whereas RSV and ADV showed less distinct seasonal patterns.

### Gender distribution of virus positivity for respiratory pathogens

Among the 39 076 positive specimens, 19 586 were from males and 19 490 from females, yielding a male-to-female ratio of ∼1.005:1. Overall positivity rates did not differ significantly between sexes (χ2 = 2.5, *P* = 0.1). However, significant sex differences were observed for Flu B (χ2 = 6.0, *P* = 0.014) and ADV (χ2 = 21.1, *P* < 0.001) (Table 1). Females showed a slightly higher Flu B positivity rate, whereas males showed a significantly higher ADV positivity rate.

However, after adjusting for age group and study period in multivariable logistic regression models, no significant sex differences remained for any pathogen (Flu A: aOR = 1.02, 95% CI: 0.97–1.06, *P* = 0.460; Flu B: aOR = 0.99, 95% CI: 0.95–1.04, *P* = 0.772; ADV: aOR = 0.99, 95% CI: 0.94–1.03, *P* = 0.544; RSV: aOR = 1.01, 95% CI: 0.97–1.06, *P* = 0.542) (Table 4). These findings suggest that the univariable sex differences observed for Flu B and ADV were largely attributable to confounding by age or period effects.

### Age distribution of virus positivity for respiratory pathogens

**Figure 4:**
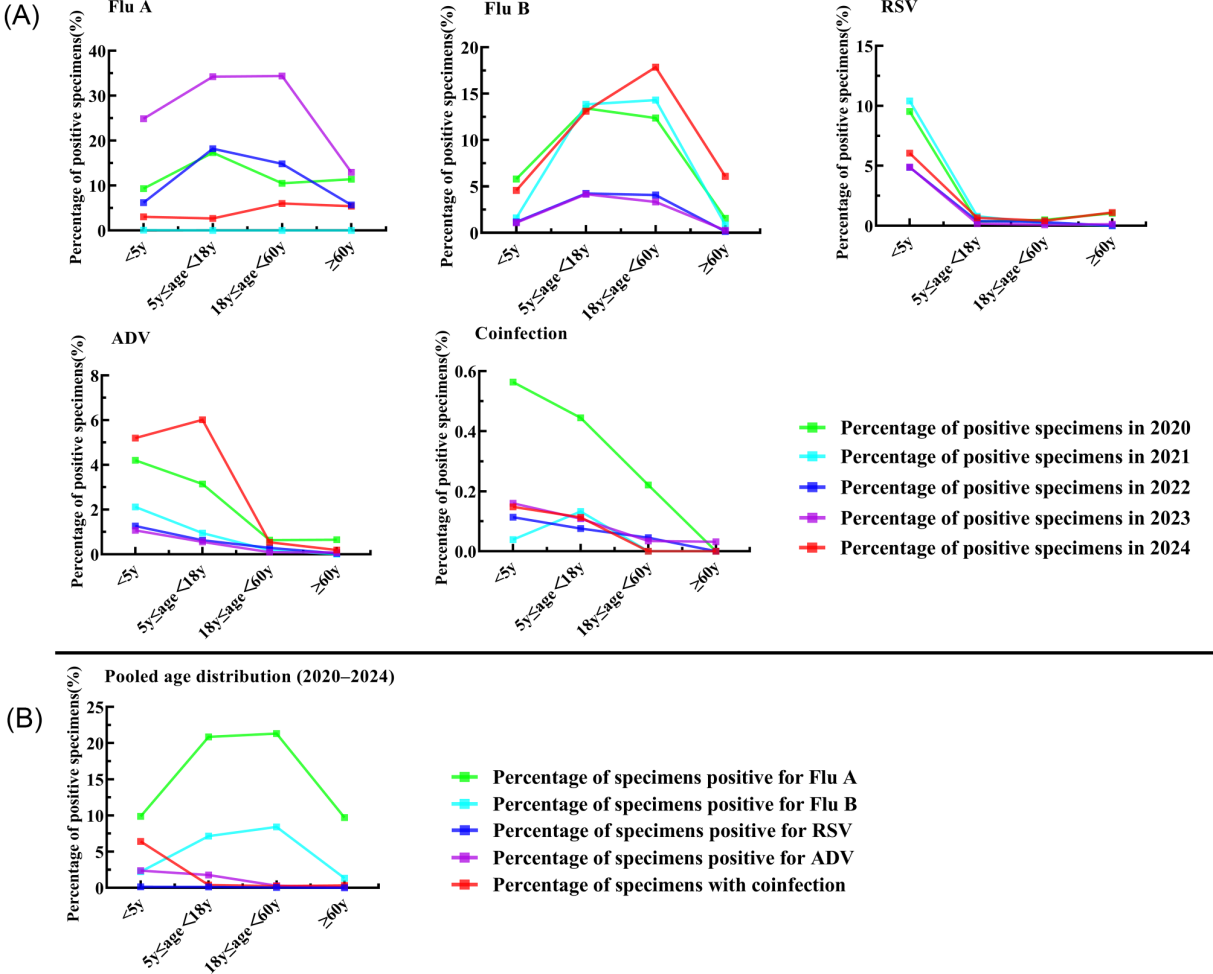
Age-stratified trends of four major respiratory viruses and coinfections from 2020 to 2024. (A) Age-stratified distribution of positivity rates by year (2020–2024). Subpanels show Flu A, Flu B, RSV, ADV, and coinfection. Each line represents one study year. (B) Pooled age-stratified distribution (2020–2024). Each line represents the pooled age-stratified distribution of positivity rates across the entire 5-year study period for Flu A, Flu B, RSV, ADV, and coinfections.

As shown in Fig. 4, positivity rates varied across years and age groups. Overall, Flu A and Flu B positivity rates were highest in adults aged 18–60 years (21.3% and 8.4%), followed by the 5–18 age group (20.9% and 7.2%). RSV, ADV, and coinfections were most common in children aged <5 years (6.4%, 2.4%, and 0.2%), followed by the 5–18 age group (0.4%, 1.8%, and 0.1%) (Table 5). From 2020 to 2021, the 5–18 and 18–60 age groups were the main affected populations. From 2022 to 2024, infections were concentrated mainly in the <5 and 5–18 age groups. RSV, ADV, and coinfection positivity rates decreased with age, whereas Flu A and Flu B showed fluctuating age-related trends.

**Table 5:** Comparison of the age distribution of the number and positivity rates of five types of viral infections from 2020 to 2024.

	2020	2021	2022	2023	2024	Total	*P* value
**Flu A**							
Median(min,max)	12y (5 m,93y)	5y (5y,8y)	9y (2 m,92y)	15y (1 m,97y)	28y (3 m,92y)	14y (1 m,97y)	
<5y	182/1 952	4/5 143	491/7 928	2 023/8 132	186/6 078	2 886/29 233	<0.001*
	(9.3%)	(0.1%)	(6.2%)	(24.9%)	(3.1%)	(9.9%)	
5y≤age<18y	546/3 152	1/4 525	2 404/13 218	8 539/24 950	283/10 604	11 773/56 449	<0.001*
	(17.3%)	(0)	(18.2%)	(34.2%)	(2.7%)	(20.9%)	
18y≤age<60y	381/3 628	0/538	984/6 636	8058/23 449	830/13 831	10 253/48 082	<0.001*
	(10.5%)	(0)	(14.8%)	(34.4%)	(6.0%)	(21.3%)	
≥60y	88/771	0/234	150/2 641	820/6 334	113/2 086	1 171/12 066	<0.001*
	(11.4%)	(0)	(5.7%)	(13.0%)	(5.4%)	(9.7%)	
**Flu B**							
Median(min,max)	13y (4 m,88y)	8y (7 m,79y)	10y (4 m,68y)	14y (3 m,89y)	27y (2 m,97y)	16y (2 m,97y)	
<5y	113/1 952	82/5 143	92/7 928	88/8 132	278/6 078	653/29 233	<0.001*
	(5.8%)	(1.6%)	(1.2%)	(1.1%)	(4.6%)	(2.2%)	
5y≤age<18y	423/3 152	626/4 525	560/13 218	1 037/24 950	1 391/10 604	4 037/56 449	<0.001*
	(13.4%)	(13.8%)	(4.2%)	(4.2%)	(13.1%)	(7.2%)	
18y≤age<60y	449/3 628	77/538	270/6 636	779/23 449	2 469/13 831	4 044/48 082	<0.001*
	(12.4%)	(14.3%)	(4.1%)	(3.3%)	(17.9%)	(8.4%)	
≥60y	12/771	2/234	4/2 641	17/6 334	127/2 086	162/12 066	<0.001*
	(1.6%)	(0.9%)	(0.2%)	(0.3%)	(6.1%)	(1.3%)	
**RSV**							
Median(min,max)	2y (2 m,82y)	2y (1 m,29y)	3y (1 m,59y)	3y (1 m,83y)	3y (1 m,95y)	3y (1 m,95y)	
<5y	186/1 952	535/5 143	386/7 928	398/8 132	368/6 078	1 873/29 233	<0.001*
	(9.5%)	(10.4%)	(4.9%)	(4.9%)	(6.1%)	(6.4%)	
5y≤age<18y	11/3 152	36/4 525	52/13 218	45/24 950	70/10 604	214/56 449	<0.001*
	(0.4%)	(0.8%)	(0.4%)	(0.2%)	(0.7%)	(0.4%)	
18y≤age<60y	18/3 628	1/538	20/6 636	18/23 449	57/13 831	114/48 082	<0.001*
	(0.5%)	(0.2%)	(0.3%)	(0.1%)	(0.4%)	(0.2%)	
≥60y	8/771	0/234	0/2641	8/6 334	23/2 086	39/12 066	<0.001*
	(1.0%)	(0)	(0)	(0.1%)	(1.1%)	(0.3%)	
**ADV**							
Median(min,max)	5y (8 m,88y)	3y (5 m,35y)	5y (6 m,91y)	6y (1 m,79y)	6y (4 m,92y)	6y (1 m,92y)	
<5y	82/1 952	109/5 143	100/7 928	87/8 132	316/6 078	694/29 233	<0.001*
	(4.2%)	(2.1%)	(1.3%)	(1.1%)	(5.2%)	(2.4%)	
5y≤age<18y	99/3 152	43/4 525	83/13 218	139/24 950	638/10 604	1 002/56 449	<0.001*
	(3.1%)	(1.0%)	(0.6%)	(0.6%)	(6.0%)	(1.8%)	
18y≤age<60y	23/3 628	1/538	19/6 636	22/23 449	74/13 831	139/48 082	<0.001*
	(0.6%)	(0.2%)	(0.3%)	(0.1%)	(0.5%)	(0.3%)	
≥60y	5/771	0/234	1/2641	5/6334	4/2 086	15/12 066	<0.001*
	(0.7%)	(0)	(0)	(0.1%)	(0.2%)	(0.1%)	
**Coinfection**							
Median(min,max)	6y (2y,40y)	6.5y (2y,10y)	5y (1y,32y)	7y (10 m,78y)	5y (8 m,7y)	7y (6 m,78y)	
<5y	11/1 925	2/5 143	9/7 928	13/8 132	9/6 078	44/29 233	
	(0.6%)	(0)	(0.1%)	(0.2%)	(0.2%)	(0.2%)	
5y≤age<18y	14/3 152	6/4 525	10/13 218	27/24 950	12/10 604	69/56 449	
	(0.4%)	(0.1%)	(0.1%)	(0.1%)	(0.1%)	(0.1%)	
18y≤age<60y	8/3 628	0/538	3/6 636	8/23 449	0/13 831	19/48 082	
	(0.2%)	(0)	(0.1%)	(0)	(0)	(0)	
≥60y	0/771	0/234	0/2 641	2/6 334	0/2086	2/12 066	
	(0)	(0)	(0)	(0)	(0)	(0)	

Abbreviations: Flu A, influenza A; Flu B, influenza B; RSV, respiratory syncytial virus; ADV, adenovirus. *P < 0.05.

Multivariable logistic regression confirmed that older age groups had significantly higher adjusted odds of positivity compared with children <5 years across all four pathogens (Table 4). Adults aged 18–60 years exhibited the highest adjusted odds (Flu A: aOR = 2.86, 95% CI: 2.55–3.20; Flu B: aOR = 2.40, 95% CI: 2.15–2.69; ADV: aOR = 2.33, 95% CI: 2.08–2.61; RSV: aOR = 2.06, 95% CI: 1.85–2.29; all *P* < 0.001).

### Age-stratified logistic regression and period-age interactions

To examine whether the effect of NPI relaxation differed across age strata, we performed age-group-stratified logistic regression analyses for each pathogen, adjusting for sex (Table 6). The lower adjusted odds of positivity during the post-relaxation period were consistent across all age groups for all four pathogens. However, the magnitude of this reduction increased with age. For Flu A, the aORs were 0.63 (95% CI: 0.56–0.71) for children <5 years, 0.71 (95% CI: 0.66–0.76) for school-age children, 0.58 (95% CI: 0.52–0.64) for adults, and 0.41 (95% CI: 0.31–0.55) for geriatric populations (all *P* < 0.001). Similar age-dependent patterns were observed for Flu B, ADV, and RSV (Table 6).

**Table 6:** Adjusted odds ratios for respiratory virus positivity during the post-relaxation period, stratified by age group.

Pathogen	Age group	aOR (95% CI)	*P*
**Flu A**	<5y	0.63 (0.56–0.71)	<0.001
	5y≤age<18y	0.71 (0.66–0.76)	<0.001
	18y≤age<60y	0.58 (0.52–0.64)	<0.001
	≥60y	0.41 (0.31–0.55)	<0.001
**Flu B**	<5y	0.77 (0.68–0.87)	<0.001
	5y≤age<18y	0.69 (0.64–0.74)	<0.001
	18y≤age<60y	0.62 (0.56–0.69)	<0.001
	≥60y	0.38 (0.28–0.50)	<0.001
**RSV**	<5y	0.84 (0.75–0.95)	0.003
	5y≤age<18y	0.56 (0.52–0.61)	<0.001
	18y≤age<60y	0.61 (0.56–0.68)	
	<0.001		
	≥60y	0.44 (0.34–0.57)	
	<0.001		
**ADV**	<5y	**0.71 (0.63–0.80)**	<0.001
	5y≤age<18y	**0.72 (0.66–0.78)**	<0.001
	18y≤age<60y	**0.74 (0.68–0.82)**	<0.001
	≥60y	**0.62 (0.48–0.80)**	<0.001

aOR = adjusted odds ratio; CI = confidence interval. Multivariable logistic regression models were stratified by age group and adjusted for sex. The strict NPI period (2020–2022) served as the reference category for period; female served as the reference category for sex.

## Discussion

Since the COVID-19 pandemic, countries worldwide have implemented NPIs, including travel restrictions, mask wearing, social distancing, hand hygiene, and home isolation, to reduce viral transmission. In China, strict containment measures were initially adopted and later transitioned into a “dynamic zero-COVID” policy until December 2022, after which restrictions were gradually relaxed and social activities resumed (Ge et al. 2023).

**Figure 5:**
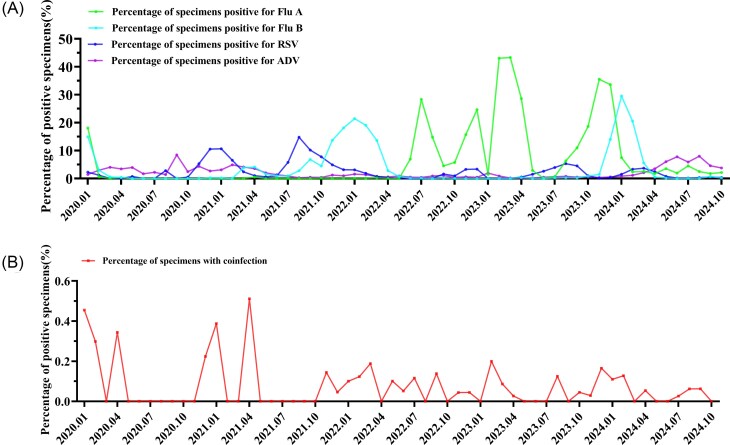
Epidemiological trends of four respiratory pathogens and coinfections from 2020 to 2024. (A) Temporal trends in the prevalence of four respiratory pathogens and coinfections from 2020 to 2024. (B) Temporal trends in the prevalence of coinfections from 2020 to 2024.

This retrospective study evaluated the epidemiological dynamics of four major respiratory pathogens in Hangzhou, China, during the COVID-19 pandemic (January 2020–December 2022) and after the relaxation of restrictions (January 2023-October 2024). During strict NPIs, overall positivity rates declined sharply from 27.4% in 2020 to 14.5% in 2021 (Fig. 5A), consistent with global reports of reduced respiratory virus transmission under stringent control measures (Olsen et al. 2021). Reduced viral circulation may also have lowered population immunity, increasing susceptibility after restrictions were lifted. Positivity rates rebounded to 18.5% in 2022 and 35.1% in 2023 before declining to 22.2% in 2024, suggesting a rebound effect associated with waning immunity and renewed viral circulation (Paireau et al. 2023). Increased specimen collection in 2023, partly due to respiratory virus monitoring programs in primary and secondary schools, may also have contributed to the higher positivity rate. During the strict NPI period, testing was largely restricted to symptomatic inpatients and outpatients with severe ARIs, yielding a high pre-test probability and, consequently, elevated crude positivity. After relaxation, expanded school-based screening and community surveillance substantially broadened the tested denominator to include milder and asymptomatic cases, mechanically diluting the crude positivity rate. Multivariable logistic regression, which adjusts for these concurrent demographic shifts, provides a less confounded estimate of the true period effect. The consistently lower adjusted odds of positivity during the post-relaxation period (aORs 0.64–0.72) suggest that, after accounting for changes in the tested population, the intrinsic risk of viral detection was actually lower in 2023–2024 than during 2020–2022. This finding underscores the importance of adjusting for demographic confounders when comparing positivity rates across periods with differing testing policies. Therefore, in addition to the relaxation of the epidemic-related restrictions, changes in testing policies, healthcare-seeking behavior, outpatient volume, and hospital admission thresholds may likewise have influenced positivity rates.

Figure 5 demonstrates divergent trends among respiratory pathogens and coinfections from 2020 to 2024. Flu A and Flu B became the dominant pathogens during the winter-spring seasons of 2023–2024, with coinfections also peaking during this period (Fig. 5B), consistent with findings from other regions (Cai et al. 2024). ADV positivity increased during 2022–2024 compared with 2020–2021, whereas RSV positivity declined after 2021 before rising again, consistent with previous reports (Zhao et al. 2024). These findings suggest virus-specific differences in resilience to public health interventions (Quintero-Salgado et al. 2024). Overall, these divergent trajectories underscore that the observed shifts reflect a confluence of factors-including virus-specific biological resilience, altered population immunity profiles, and concurrent changes in testing intensity and healthcare-seeking behavior-rather than the direct, uniform effect of NPIs relaxation alone.

Before the COVID-19 pandemic, influenza epidemics generally followed stable seasonal patterns (Chadha et al. 2020). In Hangzhou’s subtropical monsoon climate, Flu A showed dual peaks in spring (23.4%) and winter (18.0%), whereas Flu B peaked mainly in winter (16.1%). These findings are consistent with influenza transmission patterns in subtropical regions (Petrova et al. 2018). In 2021, influenza activity remained extremely low, with only 5 Flu A cases detected (0.1%), similar to reports from Shijiazhuang, where only 2 cases were identified in the same year (Zhao et al. 2024). Previous models predicted influenza resurgence after resumption of social activities because of reduced population exposure and increased susceptibility during the pandemic (Baker et al. 2020). Our 2022–2023 data support this prediction, although the rebound likely reflected a combination of immunological susceptibility, expanded testing capacity, and resumed social mixing, rather than NPI relaxation as the sole driver, although the limited number of coinfections prevented further analysis.

Before the pandemic, RSV in China typically peaked during winter (Guo et al. 2024). In this study, RSV seasonality was weaker (χ² = 227.7), with peaks shifting from autumn (2020–2021) to winter (2022), followed by an atypical summer peak in 2023 before returning to winter-spring peaks in 2024. Similar atypical seasonal patterns have been reported internationally (Casalegno et al. 2021, Ohnishi et al. 2021, Chiu et al. 2022, Nenna et al. 2022). In addition to NPIs disrupting viral circulation, the emergence of new viral lineages may also have contributed to these changes.

NPIs also affected the seasonal prevalence of ADV. From 2020 to 2023, ADV showed irregular seasonal patterns, with low positivity rates and varying epidemic peaks across seasons (Table 2). Strict NPIs in early 2020, including social distancing, mask wearing, and school closures, likely suppressed ADV transmission. By 2024, ADV seasonality returned to prepandemic patterns, characterized by summer peaks (Zhu et al. 2021), consistent with the findings of Zhou H et al. (Zhou et al. 2024). Increased travel and social interaction after relaxation of NPIs may have contributed to this recovery. In contrast to the age-dependent period effects observed for influenza viruses, ADV showed a remarkably homogeneous reduction across age strata (aORs 0.71–0.74, Table 6), suggesting that NPI relaxation influenced ADV epidemiology through population-level mechanisms-such as increased school and community mixing-that affected all age groups similarly.

Univariable analysis suggested marginally higher Flu B positivity among females and higher ADV positivity among males; however, these differences disappeared entirely after adjusting for age group and study period in multivariable logistic regression models (all *P* > 0.05). This finding indicates that the observed sex differences in crude rates were largely attributable to confounding by age structure and period effects-specifically, differences in the demographic composition of the tested populations between the NPI and post-relaxation eras rather than true biological susceptibility (Tadount et al. 2020). Indeed, sex is recognized as a common confounder in respiratory infection epidemiology, and multivariable adjustment is required to disentangle its independent effect from concurrent demographic shifts (Ursin et al. 2021). Consequently, our data do not support sex as an independent predictor of positivity for these pathogens once demographic shifts are accounted for. This aligns with the broader understanding that apparent sex disparities in respiratory infection rates often reflect differential exposure patterns, healthcare-seeking behavior, or testing access rather than intrinsic immunological differences (Binu et al. 2026), a pattern similarly observed in studies where sex lost significance after adjustment for clinical severity and care-seeking behavior.

Age-stratified analyses revealed the most nuanced dimension of epidemiological shifting. Adults aged 18–60 years maintained the highest absolute adjusted odds across both periods (aORs 2.06–2.86, Table 4), confirming their status as the core at-risk population. However, the magnitude of period-related reduction was greatest in geriatric populations and adults (Flu A aORs 0.41–0.58), whereas school-age children showed relatively attenuated declines (aOR 0.71). ADV exhibited a homogeneous reduction across ages (aORs 0.71–0.74), suggesting population-level mixing effects, while RSV in children <5 years demonstrated the weakest period effect (aOR = 0.84, 95% CI: 0.75–0.95) (Li et al. 2022), underscoring the limited impact of NPIs on RSV transmission in this highest-risk cohort. By 2024, crude influenza positivity appeared to concentrate in adults and geriatric populations, but this shift reflected expanded testing into adult populations rather than a genuine redistribution of disease burden. The adjusted models indicate that the intrinsic odds in adults, while highest in absolute terms, had declined proportionally more than in younger cohorts after NPIs relaxation. These age-dependent shifts highlight that the postpandemic epidemiological landscape is characterized not by a uniform resurgence across all age groups, but by differentially attenuated period effects superimposed on stable age-specific risk hierarchies.

From a public health perspective, these findings redefine how postpandemic respiratory virus resurgence should be interpreted. First, crude positivity rates are inadequate metrics for evaluating resurgence; without adjusting for demographic and testing-policy shifts, policymakers risk conflating denominator expansion with true transmission surges (Chartrand et al. 2012). Second, the heterogeneous age-pattern of period effects-with adults showing the greatest relative reduction despite highest absolute risk-suggests that adaptive vaccination and surveillance strategies should prioritize adults and geriatric populations for influenza, while sustained pediatric prevention remains critical for RSV and ADV (Fairchok et al. 2020). These insights support data-driven, age-specific public health strategies tailored to the shifting postpandemic epidemiology of Hangzhou and similar subtropical Chinese cities.

Several limitations should be acknowledged. First, the single-center, retrospective design may limit generalizability beyond Hangzhou. Second, the absence of vaccination history precluded assessment of vaccine effectiveness. Third, surveillance relied solely on colloidal gold antigen detection, which has lower sensitivity than real-time RT-PCR, especially in low viral-load samples (Centers for Disease Control and Prevention 2026), potentially underestimating true viral circulation. Fourth, seasonal variation was not adjusted for in multivariable models; because the post-relaxation period included incomplete calendar years (through October 2024) and respiratory viruses exhibit strong seasonality (Li et al. 2019), residual confounding by season cannot be excluded. Fifth, classifying 2022 entirely into the strict NPI period represents a simplification, as relaxation began in late December 2022. Finally, multiomics approaches may further clarify the complex interactions among host, pathogen, and environmental factors that influence respiratory virus transmission in the post-NPI era.

## Conclusions

This study demonstrates that the postpandemic resurgence of respiratory viruses in Hangzhou (2020–2024) was not a uniform rebound following NPI relaxation, but a shifting epidemiology shaped by virus-specific resilience, expanded testing policies, and changing demographic denominators. Multivariable models revealed lower intrinsic odds of detection after restriction easing, indicating that crude positivity surges partly reflected surveillance structural shifts. Age-stratified analyses further showed heterogeneous period effects superimposed on stable risk hierarchies, with RSV and ADV persisting in children and influenza dominating in adults. These findings support adaptive, age-targeted public health strategies that account for both biological transmission dynamics and surveillance artifacts in the post-COVID-19 era.

## Supplementary Material

xtag040_Supplemental_File

## Data Availability

Statement Additional data are available from the first author and corresponding author upon reasonable request.
