# Supplementary material for: Nonpharmaceutical interventions and postpandemic resurgence: the shifting epidemiology of influenza, respiratory syncytial virus, and adenovirus in Hangzhou, Zhejiang Province (2020–2024)
Source: FEMS Microbes. 2026 Jul 17;7:xtag040. doi: 10.1093/femsmc/xtag040 (PMC13436969; doi:10.1093/femsmc/xtag040)
Supplement: Supplementary file 1 [file xtag040_supplemental_file.docx]

**Reviews of manuscript FEMSMC-2026-011**

**Reviewer 1: Qianhui Wu, Fudan University**

**Recommendation: Major Revision**

The authors conducted a large-scale hospital-based surveillance study of four common respiratory viruses (Influenza A, Influenza B, RSV, and ADV) detected in patients with acute respiratory infection (ARI) in Hangzhou, China, from 2020 to 2024. The dataset was large and potentially valuable, especially in documenting the four respiratory pathogen activities across the COVID-19 and post-restriction periods.

On that basis, I would like to raise my comments as follow:

Major Comments

Overall

1. The authors should revise the language carefully to improve clarity and coherence. There are multiple grammatical errors, typographical inconsistencies and unclear expressions.

2. The Results and Discussion sections should be reorganized to improve logical flow and clarity. They would benefit from being more concise and more clearly focused on the primary findings and key conclusions, avoiding tangential or loosely connected content.

3. There are multiple instances of misplaced content throughout the manuscript. The authors should carefully review the entire text to identify sections that are out of place and reorganize the manuscript to ensure a clear, coherent, and logically structured presentation.

In the Introduction Section,

1. Line 58, the phrase “most etiologically confirmed cases” lacks supporting quantitative data, and the four cited references do not fully align with the statement - three were conducted during or post COVID-19 period in China, and one is a global modeling study on influenza mortality, which does not directly support the claim.

2. Abbreviations. RSV and ADV are introduced in Line 59 before their full names appear in Lines 70 and 72.

3. The sentence in Line 68 (“which was associated In 2019”) is grammatically incomplete and lacks logical continuity, and the phrase “While shifts in respiratory pathogen” in Line 75 does not connect coherently with the surrounding text.

4. The data were derived solely from a tertiary hospital in Hangzhou, the authors should avoid overgeneralizing the findings to the “provincial-specific” (Line 81) without adequate justification.

In the Method Section,

1. Were all eligible outpatients and inpatients tested and included in the analysis, or was a subset selected? If so, the selection method and proportion should be specified. And the authors should clarify why the sample size in 2023 was substantially larger than in other years.

2. Given the strict control measures and region-based testing responsibilities in China during 2020-2022, the source population may differ from that in 2023, when healthcare-seeking patterns likely returned to routine. The authors should clarify whether the study populations before and after 2022 were comparable.

3. The statistical analysis relied exclusively on Pearson’s chi-square and Fisher’s exact tests. Given the very large sample size, statistical significance is almost guaranteed even for small effect sizes. For a multi-year epidemiological dataset of this scale, more appropriate analyses may include, such as logistic regression models, stratified or multivariable analyses adjusting for age and sex. Corresponding effect sizes and confidence intervals should be reported.

In the Results Section,

1. Line 158, the interpretation regarding subtype predominance (H3N2) should be removed, as the antigen detection kit used in this study does not allow for influenza subtyping (if could, clarify in Method Section).

2. Lines 166-170, comparisons with national surveillance data and recent studies should be moved to the Discussion section, as they are more appropriate for interpretation rather than presentation of results.

3. Lines 2023-213, the content does not match the subheading. Moreover, it is unclear why content related to COVID-19 appears here (Lines 207-210).

4. Table 1-3, The interpretation of the statistical findings is currently insufficient. Further clarification is needed.

5. Figures 2-4, the visualization could be further improved. Currently, each panel contains too many colors and symbols, which makes the figures difficult to interpret. Simplifying the graphical elements would enhance clarity. The authors should consider removing the “the number of positive specimens” if they do not add meaningful insight beyond the “percentage of positivity specimens”.

In the Discussion Section,

1．As mentioned in Line 77, “similar research has been conducted in Shanghai”, which is geographically close to Hangzhou, Zhejiang. However, no comparison with these findings is provided in the Discussion.

2. Lines 237-252 contain descriptions of the study findings and may be better placed in the Results section.

3. The authors overinterpreted the impact of non-pharmaceutical interventions (NPIs) on seasonal prevalence of ADV and RSV. The study does not account for changes in testing policies, changes in healthcare-seeking behavior, variations in total outpatient volume and changes in hospital admission thresholds. Without adjustment for these factors, causal interpretations should be softened. The conclusions should be reframed as observational associations rather than causal inferences.

Minor Comments

1. For clarity and consistency with common usage, the authors may consider replacing “geriatric populations” (Line 57) with “older adults”, “SPSS version 26.0 has been used” should be “was used”, “P > 0.05 indicating no significance” should be “not statistically significant.”

**Reviewer 2**

**Recommendation: Reject**

Thank you for the invitation for reviewing this paper. The paper by Zhang et al. depicted the changes in the activity of four respiratory viruses during and post COVID-19 pandemic time and compared it with the pre-pandemic periods. However, there are a few key issues, and my main recommendation is rejection, as the manuscript and the analyses required further improvement.

Major comments:

One of the major issues of the paper is the language. Throughout the paper, many sentences might need to be rephrased and some sentences were incomplete. It might be necessary to conduct proofreading before the resubmission. Several examples are as follows:

• (line 43) might reconsider using “highly statistically significant”.

• (line 67) can delete “, an orthopeumovirus”, as this paper is not focusing on the biological aspect of RSV.

• (line 68) “which was associated …” this is an incomplete sentence.

• (line 70) can delete “as reported by The Lancet”.

• (line 72) might need to clarify what the “cases linked to ADV” represents (e.g., ADV-positive cases?)

• (line 77) might need to rephrase the sentences “however, knowledge deficits….”

• (line 133) “A Chi-square test…” this belongs to the methods section, and it is unnecessary to report the detailed value of the chi-square test (line 135).

• (line 237-243, line 253) The description of the results should be in the results section instead of discussion section.

Another major issue is how the results were presented. The current presentation of the results is also a bit hard to read, especially the tables. For example, in Figure 2, it might be clearer if the histograms were removed. The information provided by the number of positive cases might not be that informative compared to the positivity rate. For Figure 3, as many colors have already been included, it is hard to distinguish the shape of the points.

It is also unclear about why some of the analyses were conducted and the main goal of the study. As stated in the introduction, similar study has been conducted in other cities and results have shown similar changes in the viral activity, it is therefore unclear why a similar study should be done in another city. It might require further clarification on the mentioned “knowledge gap” mentioned in the introduction section (line 78). Similarly, it also lacks clarification for the analysis by gender, as gender might not be an important factor in the change of viral activity.

Other minor issues:

• (line 91) what is the reason excluding those aged under 28 days?

• (line 133) maybe can keep only one decimal place in the result (similar for other estimates in the results section).

**Associate Editor Recommendation: Major Revision**

**EIC Decision: Major Revision**

**Author's Response to Decision Letter for (FEMSMC-2026-011)**

Dear reviewer,

We sincerely thank the reviewers for the valuable time and insightful comments provided on our manuscript. We have carefully considered all points raised and have revised the manuscript accordingly. The revisions have significantly improved the clarity, rigor, and overall quality of our work.

A point-by-point response to the reviewers' comments is provided below. All changes in the manuscript have been highlighted for your convenience.

Response to Reviewers' Comments

Reviewer:1

We are grateful for the thorough and constructive feedback. We have addressed all major and minor comments as detailed below.

Major Comments:

Overall:

1. Language and Clarity:The entire manuscript was revised through the services of Enago, as recommended, and revised by a native English-speaking scientific editor to correct grammatical errors, improve sentence structure, and enhance overall coherence and clarity.

2. Reorganization of Results and Discussion: We have extensively restructured these sections. The Results section is now strictly focused on presenting our primary findings concisely, removing interpretive comparisons. The Discussion has been reorganized to first summarize key results, then logically interpret them in the context of existing literature, address limitations, and state conclusions, avoiding tangential content.

3. Manuscript Flow and Content Placement: We have conducted a full review and reorganization. Content has been relocated to its appropriate section (e.g., comparisons with national data moved from Results to Discussion; misplaced COVID-19 content removed from the demographics analysis).

Introduction:

1. Line 58 :The statement has been revised to more accurately reflect the cited literature and avoid overgeneralization, the revised text now appears in Page 4.

2. Abbreviations (RSV, ADV):The abbreviations have been introduced correctly, with full names appearing first before subsequent use.

3. Grammar and Logic (Lines 68, 75): The incomplete sentence has been removed. The connection of the phrase in Line 75 has been clarified within its paragraph.

4. Overgeneralization (Line 81):We have tempered our language. References to "provincial-specific" patterns have been qualified to accurately reflect the single-center nature of our study, , the revised text now appears in Line 106, Page 5.

Methods:

1. Sample Selection and 2023 Size: We have clarified that all eligible patients meeting the clinical case definition were tested. Increased specimen collection in 2023, partly due to respiratory virus monitoring programs in primary and secondary schools, and it might also be attributed to the relaxation of NPIs, which contributed to the higher number of tests, the revised text now appears in Lines250-254, Page 10.

2. Statistical Analysis: As recommended, we have performed additional analyses.

We fully agree with this critical assessment, and this comment was the primary driver of our major revision. The original exclusive reliance on chi-square and Fisher's exact tests was indeed inadequate for a dataset of this magnitude (n = 145,936), as these methods cannot adjust for confounders or distinguish clinically meaningful effects from statistically significant but trivial associations. We have completely overhauled the statistical analysis and rewritten the Results and Discussion accordingly.

In the revised Methods (Section 2.3), we have added “To evaluate the independent effect of pandemic policy relaxation (strict NPI period [2020-2022] vs. post-relaxation period [2023-2024]) on virus positivity while adjusting for demographic shifts between periods, multivariable binary logistic regression models were constructed for each pathogen, with age group and sex as covariates. Age-group–stratified analyses were also performed to examine potential effect modification by age. Given the large sample size, we emphasized effect sizes (adjusted odds ratios [aORs] with 95% confidence intervals [CIs]) over P values. Statistical significance was set at *P* < 0.05."

In the revised Results (Section 3.6) and Discussion (Paragraph 6, 8), we frame this shift as the central confounding factor of the study. Rather than treating the two periods as demographically equivalent, we explicitly state that direct crude comparisons are invalid. The multivariable binary logistic regression models (Table 4) were specifically constructed to adjust for these concurrent demographic shifts (age group and sex) when estimating period effects. We further caution in the Discussion that residual confounding from unmeasured factors—such as healthcare-seeking behavior, outpatient volume, and hospital admission thresholds—precludes definitive causal inference, and findings should be interpreted as adjusted observational associations within a natural experiment framework.

Results:

1. Line 158 (Influenza Subtyping):The interpretation regarding H3N2 predominance has been removed.

2. Lines 166-170 (National Data Comparison): This content has been moved to the Discussion section.

3. Lines 202-213 (Content Mismatch and COVID-19): The subheading has been corrected to match the content (now focusing on demographic distributions). The unrelated COVID-19 sentences have been removed.

4. Tables 1-3 (Statistical Interpretation): The tables and corresponding text have been revised, providing a clearer measure of association strength.

5. Figures 2-4 (Visualization): All figures have been simplified per the reviewer's suggestion. The "number of positive specimens" line and secondary Y-axis have been removed. The graphs now display only the monthly positivity percentage for each virus, using a clearer color scheme and legend, significantly improving readability.

Discussion:

1. Comparison with Shanghai Study: A new paragraph has been added to compare and contrast our key findings (e.g., summer influenza peak, RSV seasonality) with the cited similar study from other regions, the revised text now appears in Lines268-274,Page 10.

2. Lines 237-252 (Content Placement): This descriptive paragraph summarizing findings has been removed.

3. Overinterpretation of NPI Impact: We have significantly softened causal language regarding NPI effects. Statements have been reframed to describe "observed associations" and "epidemiological shifts coinciding with" NPI changes. The Limitations section has been strengthened to explicitly list confounding factors noted by the reviewer (testing policies, healthcare-seeking behavior, etc.), the revised text now appears in Lines312-326,Page 12.

Minor Comments: All minor points have been addressed:

1. "Geriatric populations" has been replaced with "older adults."

2. "SPSS version 26.0 has been used" is now "was used."

3. "P > 0.05 indicating no significance" is now "P values < 0.05 were considered statistically significant."

Reviewer:2

1. Line 43: highly statistically significant" has been rephrased.

2. Line 67: ", an orthopneumovirus" has been deleted.

3. Line 68: The incomplete sentence has been rewritten for clarity and grammatical correctness.

4. Line 70: "as reported by The Lancet" has been deleted.

5. Line 72: Clarified to "ADV-positive cases."

6. Line 77: The sentence "however, knowledge deficits..." has been rewritten again and the new literature has been cited.

7. Lines 133-135:The detailed chi-square test description and value in the Results section have been removed, as correctly suggested.

8. Presentation of Results and Figures: Comment: The results and figures (especially Figure 2 and 3) were hard to read. Suggestions included removing histograms and simplifying point shapes in Figure 3. Response: We appreciate this constructive feedback on data visualization. We have significantly revised all figures to enhance clarity.

9. Line 78: We have strengthened the Introduction and deleted the “the knowledge gap”. While studies exist, our work provides a detailed, longitudinal (2020-2024) analysis from a major urban center in East China, capturing the full transition from strict NPIs through to their relaxation.

10. Line 91 (Exclusion of infants <28 days): We have added a clarification in the Methods: "Age 1 day-100 years; either sex."

11. Line 133 (Decimal places): As suggested, we have standardized the presentation of percentages and statistical values to one decimal place throughout the Results section for consistency.

Thank you again for the opportunity to revise our work.

Sincerely,

Shaoni Zhang

**Reviews of manuscript FEMSMC-2026-011.R1**

**Associate Editor Recommendation: Minor Revision**

Overall the revisions are acceptable. Figures 3 and 4 are confusing a laid out. Panells A-E could have headers to make it clear which virus is shown in each without needing to refer to the legend. Panel E uses a different grouping and definitions, and it is confusing to include it with the others. Th labeling of the figures is also confusing. It is states as 'Percentage of Flu-A positive specimens' but I think it is 'Percentage of specimens that are positive for Flu-A' which is a different subset.

**EIC Decision: Minor Revision**

**Author's Response to Decision Letter for (FEMSMC-2026-011)**

Dear Associate Editor,

We sincerely thank you for the valuable time and insightful comments provided on our manuscript. A point-by-point response to associate editor's comments is provided below.

Associate Editor Comment:

1.Figures 3 and 4 are confusing as laid out.

Response: We have completely restructured figures 3 and 4 into a clean, two-panel format:

Panel A: Year-stratified trends (2020-2024) for the four primary pathogens, with clear sub-headers.

Panel B: Pooled distribution across the entire 5-year period (2020–2024), presented as a standalone panel with its own distinct analytical dimension.

2.Panells A-E could have headers to make it clear which virus is shown in each without needing to refer to the legend. Panel F uses a different grouping and definitions, and it is confusing to include it with the others.

Response: We have added explicit sub-headers to every subplot in Panel A-E. In the revised Figure 3 and Figure 4, Panel A contains five subplots labeled directly on the figure as:Flu A, Flu B, RSV, ADV, Coinfection. Panel B contains one subplot labeled directly on the figure as: Pooled seasonal distribution (2020-2024) and Pooled age distribution (2020-2024)

3.The labeling of the figures is also confusing. It is stateed as 'Percentage of Flu-A positive specimens' but I think it is 'Percentage of specimens that are positive for Flu-A' which is a different subset.

Response: We have corrected all figure labels and legends throughout the manuscript : "Percentage of Flu-A positive specimens" is now "Percentage of specimens positive for influenza A", "Percentage of RSV positive specimens" is now "Percentage of specimens positive for RSV", "Percentage of ADV positive specimens" is now "Percentage of specimens positive for ADV", "Percentage of coinfection specimens" is now "Percentage of specimens with coinfetion".

Thank you again for the opportunity to revise our work.

Sincerely,

Shaoni Zhang

**Reviews of manuscript FEMSMC-2026-011.R2**

**Associate Editor Recommendation: Accept**

**EIC Decision: Accept**
